# Obesity, Abdominal Obesity and Chronic Kidney Disease in Young Adults: A Nationwide Population-Based Cohort Study

**DOI:** 10.3390/jcm10051065

**Published:** 2021-03-04

**Authors:** Eun Hui Bae, Sang Yeob Lim, Jin-Hyung Jung, Tae Ryom Oh, Hong Sang Choi, Chang Seong Kim, Seong Kwon Ma, Kyung-Do Han, Soo Wan Kim

**Affiliations:** 1 Department of Internal Medicine, Chonnan National University Medical School, Gwangju 61469, Korea; baedak76@gmail.com (E.H.B.); tryeomoh@daum.net (T.R.O.); hongsang38@hanmail.net (H.S.C.); laminion@hanmail.net (C.S.K.); drmsk@hanmail.net (S.K.M.); 2 Department of Internal Medicine, Chonnam National University Hospital, Gwangju 61469, Korea; 3 Department of Internal Medicine, Korea University Ansan Hospital, Ansan 15459, Korea; vnlover@hanmail.net; 4 Department of Medical Statistics, College of Medicine, The Catholic University of Korea, Seoul 06591, Korea; jungjin115@naver.com; 5 Department of Statistics and Actuarial Science, Soongsil Universithy, Seoul 06978, Korea

**Keywords:** obesity, abdominal obesity, chronic kidney disease, body mass index, waist circumference, diabetes mellitus, korean

## Abstract

Obesity has become a pandemic. It is one of the strongest risk-factors of new-onset chronic kidney disease (CKD). However, the effects of obesity and abdominal obesity on the risk of developing CKD in young adults has not been elucidated. From a nationwide health screening database, we included 3,030,884 young adults aged 20–39 years without CKD during a baseline examination in 2009–2010, who could follow up during 2013–2016. Patients were stratified into five levels based on their baseline body mass index (BMI) and six levels based on their waist circumference (WC; 5-cm increments). The primary outcome was the development of CKD. During the follow up, until 2016, 5853 (0.19%) participants developed CKD. Both BMI and WC showed a U-shaped relationship with CKD risk, identifying the cut-off values as a BMI of 21 and WC of 72 cm in young adults. The obesity group (odd ratio [OR] = 1.320, 95% confidence interval [CI]: 1.247–1.397) and abdominal obesity group (male WC ≥ 90, female WC ≥ 85) (OR = 1.208, 95%CI: 1.332–1.290) showed a higher CKD risk than the non-obesity or non-abdominal obesity groups after adjusting for covariates. In the CKD risk by obesity composite, the obesity displayed by the abdominal obesity group showed the highest CKD risk (OR = 1.502, 95%CI: 1.190–1.895), especially in those under 30 years old. During subgroup analysis, the diabetes mellitus (DM) group with obesity or abdominal obesity paradoxically showed a lower CKD risk compared with the non-obesity or non-abdominal obesity group. Obesity and abdominal obesity are associated with increased risk of developing CKD in young adults but a decreased risk in young adults with diabetes.

## 1. Introduction

The prevalence of obesity has been steadily and significantly increasing worldwide [1,2]. Its impact on diabetes mellitus (DM), cardiovascular disease, and chronic kidney disease (CKD) has rendered obesity one of the most serious global health issues [3,4]. A high body mass index (BMI) is one of the strongest risk factors for new-onset CKD. Obesity is associated with incident and prevalent microalbuminuria and higher glomerular filtration rate (GFR) or hyperfiltration. Together with albuminuria, it may herald progressive kidney dysfunction and glomerulosclerosis [3,5,6]. In people with obesity, a compensatory hyperfiltration occurs to sustain the heightened metabolic demands of increased body weight. The increase in intraglomerular pressure can damage the kidneys and increase the risk of developing CKD in the long term. The incidence of obesity-related glomerulopathy has increased tenfold in recent years. Today’s young adult population is at a high risk of developing obesity-related kidney disease in the future, providing opportunities for preventive interventions [7].

Obesity is a risk factor for diabetes. However, a recent report revealed that diabetic patients who are underweight are at a higher risk of CKD [8]. This predicament suggests a need for different standards for diabetic and non-diabetic people with obesity. In addition, waist circumference (WC) appears to be a better marker of obesity-related health risks than other surrogates for obesity [5,9]. WC indicates abdominal obesity. In the era of personalized medicine, there is evidence that abdominal obesity might have a different association with CKD risk factors compared with obesity in young adults.

Therefore, we conducted this study to evaluate the relationship between obesity or abdominal obesity and CKD risk in young adults using the National Health Insurance Service’s (NHIS) health checkup data.

## 2. Experimental Section

Due to the confidentiality of the data used in this study and the strict privacy policy from the data holder indicating that the data should be kept among designated research personnel only, the data cannot be provided to others, regardless of whether or not the data are made anonymous.

### 2.1. Study Design and Database

**Figure 1:**
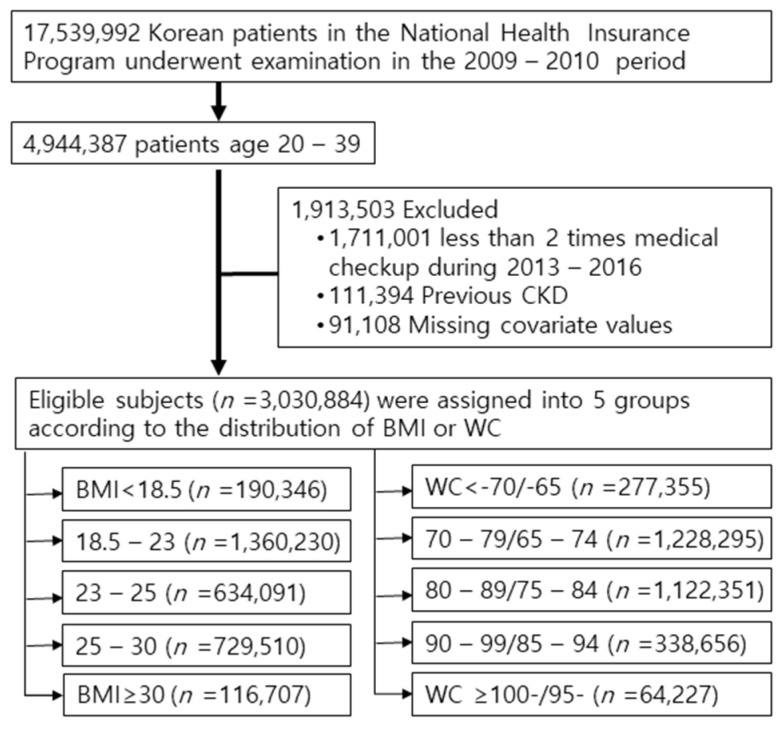
Flow diagram of the study. Abbreviations: CKD, chronic kidney disease; BMI, body mass index; WC, waist circumference.

The Korean National Health Insurance Service (NHIS) comprises a complete set of health information pertaining to 50 million Koreans, which includes an eligibility database, a medical treatment database, a health examination database, and a medical care institution database [10,11,12]. The National Health Insurance Corporation is the single insurer, managed by the Korean government, to which approximately 97% of the Korean population subscribes. Enrollees in the NHIC are recommended to undergo a standardized medical examination at least every 2 years. Among 4,944,387 young adults aged 20–39 in 2009–2010 (index year), 3,233,386 participants were selected who could follow up during 2013–2016. To avoid confounders by pre-existing diseases and minimize the possible effects of reverse causality, those who had a history of CKD before the index year were also excluded (*n* = 111,394). Ultimately, retrospective analysis was performed on 3,030,884 subjects (Figure 1).

This study was approved by the Chonnam National University Hospital (study approval number: CNUH-EXP-2020-284) and NHIS (study approval number:NHIS-2019-1-594), and it was conducted according to the principles of the Declaration of Helsinki. The need for written informed consent was waived by our review board.

### 2.2. Definitions of BMI and WC

For each participant, BMI was calculated by dividing the weight (in kg) by the square of the height (in m^2^). We defined obesity as a BMI ≥ 25 kg/m^2^. The participants were then categorized by the definition of obesity as follows: underweight (BMI < 18.5 kg/m^2^), normal (≥ 18.5 to <23 kg/m^2^), overweight (≥23 to <25 kg/m^2^), stage 1 obesity (≥25 to 30 kg/m^2^), and stage 2 obesity (≥ 30 kg/m^2^) according to the World Health Organization’s recommendations for Asian populations [13].

The WC of each participant was also measured at the midpoint between the rib cage and iliac crest by a trained examiner. The patients were divided into 6 categories based on 5-cm WC increments: < 80 cm in men and < 75 cm in women, 80–85 cm in men and 75–80 cm in women, 85–90 cm in men and 80–85 cm in women (reference group), 90–95 cm in men and 85–90 cm in women, 95–100 cm in men and 90–95 cm in women, and ≥ 100 cm in men and ≥ 95 cm in women. Abdominal obesity was defined as a WC ≥90 cm in men and ≥ 85 cm in women according to the definition of the Korean Society for the Study of Obesity [14].

### 2.3. Definition of Chronic Disease and Covariates

Type 2 DM was defined as a fasting plasma glucose level ≥ 126 mg/dL or at least one claim per year for the prescription of hypoglycemic drugs under ICD-10 codes E11–14 [15]. Patients with type 1 DM who had claims under ICD-10 code E10 were excluded from this study [16,17]. Comorbidities were identified using information gathered in the 1 year before the index date. Hypertension was defined as a previous hypertension diagnosis ICD-10 codes (I10–13, I15) and a history of taking at least 1 antihypertensive drug, or a recorded systolic blood pressure of ≥140 mmHg or diastolic blood pressure of ≥90 mmHg in the health examination database. Dyslipidemia was identified using the appropriate diagnostic code (E78) and a history of lipid-lowering drug use, or a total serum cholesterol concentration of ≥ 240 mg/dL in the health examination database. CKD was defined as an estimated glomerular filtration rate (eGFR) of < 60 mL/min/1.73 m^2^, calculated using the CKD epidemiology collaboration (CKD-EPI) equation, and as a combination of ICD-10 codes (N18–19). The participants’ fasting blood glucose (mg/dL), total cholesterol (mg/dL), triglyceride (mg/dL), high-density lipoprotein cholesterol (mg/dL), and low-density lipoprotein cholesterol (mg/dL) concentrations were measured in a fasting state. The quality of the laboratory tests has been warranted by the Korean Association for Laboratory Medicine, and the hospitals participating in the NHI health checkup programs are certified by the NHIS.

### 2.4. Study Outcomes and Follow Up

The study population was followed from baseline to the date of CKD diagnosis or until 31 December 2016. The primary end point was incident CKD, which was defined using a combination of ICD-10 codes (N18–19) and an estimated glomerular filtration rate (eGFR) of < 60 mL/min/1.73 m^2^, calculated using the CKD epidemiology collaboration equation, more than twice during 2013–2016 medical checkup.

### 2.5. General Health Behaviors and Sociodemographic Variables

Smoking history was categorized as nonsmokers, former smokers, and current smokers. Alcohol drinking was categorized into 0, 1 to ~2, or ≥ 3 times/week by frequency (none, mild, and heavy, respectively), and regular exercise, defined as vigorous physical activity for at least 20 min/day, was categorized into 0, 1 to ~4, and ≥ 5 times/week by frequency. Income level was divided by quartile: Q1 (the lowest), Q2, Q3, and Q4 (the highest).

### 2.6. Statistical Analysis

We report the mean ± SD with intervals for continuous variables and the numbers (with percentages) for categorical variables. Hazard ratios (HRs) with 95% confidence intervals (CIs) for End-stage renal disease (ESRD) in the BMI and WC categories were obtained using multivariable Cox proportional hazard models using normal BMI (BMI 18.5–23 kg/m^2^) and normal WC (85–90/80–85 cm) as references after adjustment using 3 models. Model 1 was the crude model. Model 2 was adjusted from model 1 with the addition of age, sex, income, DM, dyslipidemia, and hypertension. Model 3 was adjusted from model 2 with the addition of smoking, alcohol drinking, physical activity, and eGFR. Cumulative ESRD incidence was estimated by constructing Kaplan-Meier curves for the mean 5.4-year follow-up period, and we used the log-rank test to examine differences in ESRD development by the level of BMI and WC. We also performed subgroup analysis for DM status. A *p*-value < 0.05 was considered to reflect statistical significance. SAS version 9.3 software and SAS survey procedures (SAS Institute, Inc., Cary, NC, USA) were used for all statistical analyses.

## 3. Results

### 3.1. Baseline Characteristics of the Study Population

Table 1 shows the baseline characteristics of participants regarding the development of CKD. Among the participants, 5853 (0.19%) developed CKD. The mean age was higher among individuals who developed CKD than among those who did not (31.82 ± 4.81 vs. 35.02 ± 3.82, *p* < 0.001). The proportions of males (72.29%), people with obesity (BMI ≥ 25), and abdominal obesity (WC ≥ 90 in male, ≥ 85 in female) were higher in the incident CKD group than in the non-CKD group. Comorbidities such as diabetes, hypertension, dyslipidemia, and proteinuria were more prevalent in the CKD group than in the non-CKD group. eGFR was lower, and blood pressure (BP), total cholesterol, and glucose levels were higher in the CKD group than in the non-CKD group (Table 1). Medication rate for DM, hypertension and dyslipidemia was increased according to increasing BMI and WC (Supplementary Table S1).

Characteristics of participants classified by BMI levels and WC are presented in Table 2 and Table 3, respectively. Subjects in the underweight group (BMI <18.5) were younger, exercised less, and had a lower prevalence of DM, hypertension, and dyslipidemia (Table 2). BP, fasting glucose, and total cholesterol were also lower in the underweight group (Table 2).

CKD events were highest in those with stage 2 obesity (BMI ≥ 30). Table 3 shows that patients in the abdominal obesity group were older, mostly male, exercised less, and had a higher prevalence of DM, hypertension, and dyslipidemia (Table 3). Apart from eGFR, BP, fasting glucose, and lipid level were also higher in the abdominal obesity group (Table 3).

### 3.2. Association of BMI and WC with the Risk of CKD

**Figure 2:**
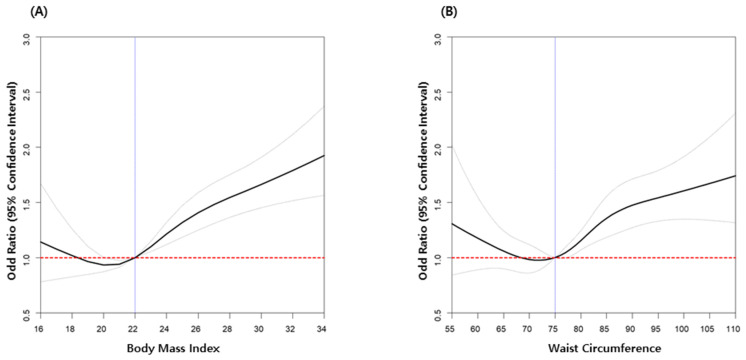
Odd ratios for chronic kidney disease according to body mass index (**A**) and waist circumference (**B**) in young adults. Adjusted for age, sex, low income (20%), diabetes mellitus, hypertension, dyslipidemia, current smoker, alcohol consumption, regular exercise, and eGFR.

CKD risk increased proportional to BMI in all participants after adjusting for age, sex, income, presence of DM, dyslipidemia, hypertension, smoking, alcohol drinking, physical activity, and GFR (Figure 2A and Table 4). The obesity group (BMI ≥ 25) showed a higher CKD risk (OR: 1.323, 95%CI: 1.250–1.400) compared with the non-obesity group (BMI < 25) (Table 4).

According to increasing WC, the risk of CKD also increased in all participants after adjusting for age, sex, income, presence of DM, dyslipidemia, hypertension, smoking, alcohol drinking, physical activity, and GFR (Figure 2B and Table 5). The abdominal obesity group (male WC ≥ 90/female WC ≥ 85) showed a higher CKD risk (OR: 1.208, 95%CI: 1.132–1.290) compared with the non-abdominal obesity group (Table 5).

Analysis for CKD risk by obesity composite showed the highest CKD risk in obesity within the abdominal obesity group (OR: 1.339, 95%CI: 1.247–1.438) compared with the only obesity (OR: 1.318, 95%CI: 1.232–1.409) or only abdominal obesity groups (OR: 1.227, 95%CI: 0.979–1.538). In addition, the abdominal-obesity-only group (OR: 1.478, 95%CI: 0.698–3.129) had higher risk of CKD compared with the obesity-only group (OR: 1.217, 95%CI: 0.968–1.531) in those under age 30 (Table 6). The correlation value between BMI and WC was 0.81441 (*p* < 0.0001)

### 3.3. Subgroup Analyses

**Figure 3:**
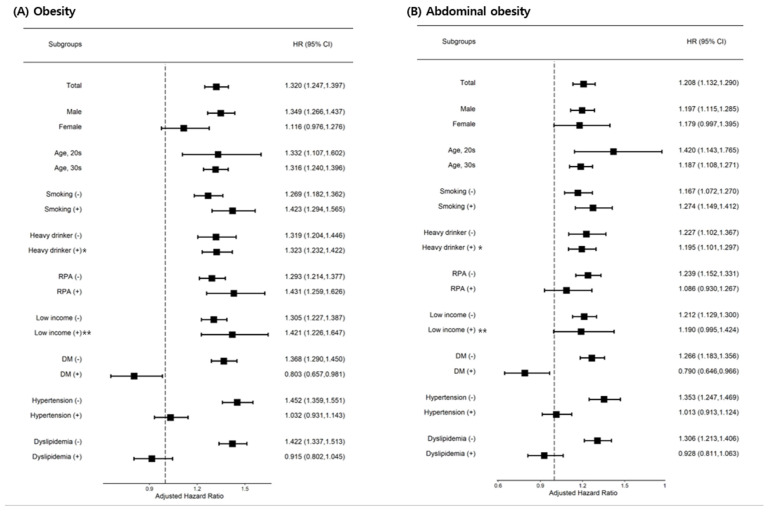
Subgroup analysis for chronic kidney disease risk. Abbreviations: * Alcohol consumptions ≥30 g/day, ** low income 20%. RPA; regular physical activity; DM, diabetes mellitus.

Subgroup analyses were performed. Female sex was not affected by obesity or abdominal obesity in the development of CKD. Abdominal obesity was not a risk factor for CKD in the regular exercise group. In the DM group, obesity and abdominal obesity had decreased risk for developing CKD. In the hypertension or dyslipidemia subgroup, CKD risk was not affected by obesity or abdominal obesity (Figure 3).

## 4. Discussion

In this large, nationwide cohort study, we demonstrated that obesity or abdominal obesity were associated with a higher risk of developing CKD in the young adult population. In addition, obesity within the abdominal obesity group showed the highest CKD risk after adjusting for potential confounders. Paradoxically, the DM group with obesity or abdominal obesity showed a lower CKD risk compared with the non-obesity or non-abdominal obesity groups in young adults.

In research settings, BMI, an internationally accepted standard anthropomorphic measurement, is generally used to define obesity [18]. Several studies have examined the association between BMI and the future risk of CKD [7,19]. Despite conflicting results, most epidemiologic studies showed that higher BMI was associated with increased risk of CKD. Recent evidence from 2015 has suggested that CKD is the second leading cause of BMI-related disability-adjusted life-years [20]. According to the Global Burden of Diseases data, mortality attributed to CKD among young adults (< 50 years) has continuously increased proportional to adolescent obesity [21]. In the United States, an annual increase of 1.6% in CKD-related mortality in young adults was recorded between 1995 and 2015. In contrast, some reports have suggested that obesity does not confer a higher risk of CKD [22] and may even play a protective role in certain settings (a phenomenon also referred to as the “obesity paradox”) [23]. In either case, most investigations into the association between obesity and CKD have been limited to obesity in young adults. Therefore, our data support a positive correlation between obesity and future CKD risk in young adults. Likewise, we provide supporting evidence for the obesity paradox occurring in the DM group in young adults.

The exact mechanism of the relationship between being underweight and increased risk of CKD remains unclear. One possible reason for this is ethnic differences. Asian people are likely to develop type 2 diabetes at a lower BMI at younger ages and suffer longer with complications [2]. Lower BMI may not necessarily guarantee health in Asian populations. The incidence of obesity-related glomerulopathy has increased tenfold in recent years [24]. Although we did not directly evaluate insulin resistance or inflammatory markers, prior studies noted that visceral adipose tissue contributes to higher circulating levels of free fatty acids and inflammatory molecules that may promote the development of insulin resistance, endothelial dysfunction, and kidney disease [25,26,27].

Measures of central or abdominal obesity, defined by WC and the waist–hip ratio, have been used recently as more important predictors for assessing mortality risk than BMI [28,29]. WC, a representative marker of visceral body fat, was found to correlate with inflammation, whereas subcutaneous body fat may be an indicator of nutritional status [30]. However, to date, there is no consensus on an appropriate definition of WC to access CKD risk because of different cut-off points for different ethnicities. In a large cohort of Japanese workers aged 20–69 years, the optimal cut-off of WC to predict later development of DM was 85 cm for men [31]. A WC of 95 cm was independently associated with a decline in eGFR (≥ 3 mL/min/1.73 m^2^/year) in men, with eGFR ranging from 60 to 90 mL/min/1.73 m^2^ [32]. A WC of ≥85 cm was more closely associated with the prevalence of CKD than a BMI of 24.0 kg/m^2^ in urban men aged 18–60 years old [33]. Our findings determined that the practical cut-off point of a WC indicating increased CKD risk was appropriately 72 cm, which is lower than previous reports. An obvious difference from previous reports is that our population consisted of healthy young adults, not common dwelling men.

The exact mechanisms explaining the association of obesity or abdominal obesity with decreasing risk of developing CKD in the young adult DM group remain uncertain, requiring further studies.

### Study Limitations

There are some limitations in this study. First, we did not collect relevant information on food habits or other comorbidities that might affect weight. Second, this study did not consider the use of medications, such as hypoglycemic agents or lipid lowing agents, and treatment adherences. Third, we were unable to obtain more information about the causes of CKD and urine protein. Fourth, we used data from the NHIS’s checkup program on a Korean population; therefore, we cannot generalize the results to other ethnic groups.

## 5. Conclusions

In conclusion, to the best of our knowledge, this is the first study on the relationship between BMI or WC and the future risk of CKD using a large general population of young adults from a well-established and validated longitudinal national database. Our study demonstrated that obesity and abdominal obesity are associated with increased risk of CKD in young adults but have a protective effect on young adults with DM.

## Figures and Tables

**Table 1:** Baseline characteristics of subjects according to the incident chronic kidney disease.

Group	Total (*n* = 3,030,884)	None CKD (*n* = 3,025,031)	CKD (*n* = 5853)	*p* Value
Age	31.83 ± 4.81	31.82 ± 4.81	35.02 ± 3.82	<0.0001
Sex, male(%)	2,030,479 (66.99)	2,026,248 (66.98)	4231 (72.29)	<0.0001
Current smoker	1,118,316 (36.9)	1,116,411 (36.91)	1905 (32.55)	<0.0001
Heavy drinker *	275,476 (9.09)	275,023 (9.09)	453 (7.74)	0.0003
Physical activity-regular	431,623 (14.24)	430,539 (14.23)	1084 (18.52)	<0.0001
Income-low **	450,211 (14.85)	449,356 (14.85)	855 (14.61)	0.596
BMI	23.22 ± 3.45	23.22 ± 3.45	24.63 ± 3.69	<0.0001
Obesity(BMI ≥ 25)	846,217 (27.92)	843,632 (27.89)	2585 (44.17)	<0.0001
BMI 5 level				<0.0001
<18.5	190,346 (6.28)	190,167 (6.29)	179 (3.06)	
18.5–23	1,360,230 (44.88)	1,358,376 (44.9)	1854 (31.68)	
23–25	634,091 (20.92)	632,856 (20.92)	1235 (21.1)	
25–30	729,510 (24.07)	727,403 (24.05)	2107 (36)	
≥30	116,707 (3.85)	116,229 (3.84)	478 (8.17)	
Waist circumference (WC)	78.42 ± 9.56	78.41 ± 9.56	81.62 ± 9.91	<0.0001
Abdominal obesity	402,883 (13.29)	401,559 (13.27)	1324 (22.62)	<0.0001
WC 5 level				<0.0001
M: <70/F: <65	277,355 (9.15)	277,050 (9.16)	305 (5.21)	
M:70–79/F:65–74	1,228,295 (40.53)	1,226,510 (40.55)	1785 (30.5)	
M:80–89/F:75–84	1,122,351 (37.03)	1,119,912 (37.02)	2439 (41.67)	
M:90–99/F:85–94	338,656 (11.17)	337,567 (11.16)	1089 (18.61)	
M: ≥100/F: ≥95	64,227 (2.12)	63,992 (2.12)	235 (4.02)	
Diabetes mellitus	58,312 (1.92)	57,867 (1.91)	445 (7.6)	<0.0001
Diabetes mellitus, vintage	0.89 ± 1.93	0.88 ± 1.91	2.86 ± 3.05	<0.0001
Hypertension	268,318 (8.85)	266,654 (8.81)	1664 (28.43)	<0.0001
Dyslipidemia	220,966 (7.29)	219,937 (7.27)	1029 (17.58)	<0.0001
CCI score	0.32 ± 0.67	0.32 ± 0.66	0.64 ± 1.1	<0.0001
CCI grade				<0.0001
0	2,303,416 (76)	2,299,590 (76.02)	3826 (65.37)	
1	561,901 (18.54)	560,852 (18.54)	1049 (17.92)	
2	120,860 (3.99)	120,310 (3.98)	550 (9.4)	
≥3	44,707 (1.48)	44,279 (1.46)	428 (7.31)	
Proteinuria				<0.0001
Negative	2,922,684 (96.67)	2,917,819 (96.7)	4865 (83.3)	
Trace	56,833 (1.88)	56,642 (1.88)	191 (3.27)	
1+	31,663 (1.05)	31,342 (1.04)	321 (5.5)	
2+	9894 (0.33)	9589 (0.32)	305 (5.22)	
3+	1872 (0.06)	1738 (0.06)	134 (2.29)	
4+	308 (0.01)	284 (0.01)	24 (0.41)	
Height (cm)	169.13 ± 8.17	169.13 ± 8.17	169.63 ± 7.86	<0.0001
Weight (cm)	66.82 ± 13.06	66.82 ± 13.06	71.3 ± 13.85	<0.0001
Fasting blood glucose (mg/dL)	91.07 ± 15.49	91.05 ± 15.43	97.71 ± 33.39	<0.0001
Systolic blood pressure (mmHg)	118.51 ± 12.88	118.5 ± 12.87	123.85 ± 15.68	<0.0001
Diastolic blood pressure (mmHg)	74.39 ± 9.27	74.38 ± 9.27	78.29 ± 11.13	<0.0001
Pulse pressure (mmHg)	44.12 ± 8.23	44.12 ± 8.23	45.55 ± 9.16	<0.0001
Total cholesterol (mg/dL)	186.42 ± 33.75	186.4 ± 33.74	197.74 ± 37.97	<0.0001
Estimated GFR (mL/min/1.73 m^2^)	96.28 ± 51.37	96.31 ± 51.36	80.26 ± 51.93	<0.0001

Abbreviations. CKD, chronic kidney disease; BMI, body mass index; WC, waist circumference; CCI, Chalson Comorbidity Index; GFR, glomerular filtration rate. * Alcohol consumptions ≥30 g/day, ** low income 25%, abdominal obesity: WC ≥ 90 in males, ≥85 in females.

**Table 2:** Baseline characteristics of participants by level of body mass index.

Variable	Distribution of Body Mass Index					
	<18.5 (*n* = 190,346)	18.5~23 (*n* = 1,360,230)	23~25 (*n* = 634,091)	25~30 (*n* = 729,510)	30~ (*n* = 116,707)	*p*
Age	29.62 ± 4.93	31.29 ± 4.94	32.41 ± 4.62	32.83 ± 4.39	32.24 ± 4.46	<0.0001
Age grade						<0.0001
20 s	101,396 (53.27)	529,544 (38.93)	184,564 (29.11)	181,446 (24.87)	33,714 (28.89)	
30 s	88,950 (46.73)	830,686 (61.07)	449,527 (70.89)	548,064 (75.13)	82,993 (71.11)	
Sex(male)	49,380 (25.94)	735,330 (54.06)	512,593 (80.84)	636,587 (87.26)	96,589 (82.76)	
Smoking						<0.0001
None	144,387 (75.86)	815,922 (59.98)	270,571 (42.67)	261,484 (35.84)	42,818 (36.69)	
Ex-	8700 (4.57)	130,974 (9.63)	100,467 (15.84)	121,109 (16.6)	16,136 (13.83)	
Current	37,259 (19.57)	413,334 (30.39)	263,053 (41.49)	346,917 (47.55)	57,753 (49.49)	
Drinking						<0.0001
None	982,92 (51.64)	556,799 (40.93)	195,454 (30.82)	203,834 (27.94)	36,666 (31.42)	
Mild	85,661 (45)	715,325 (52.59)	371,661 (58.61)	428,251 (58.7)	63,465 (54.38)	
Heavy *	6393 (3.36)	88,106 (6.48)	66,976 (10.56)	97,425 (13.35)	16,576 (14.2)	
Exercise **	12,970 (6.81)	170,635 (12.54)	105,304 (16.61)	123,655 (16.95)	19,059 (16.33)	<0.0001
Income ***	30,047 (15.79)	212,947 (15.66)	89,929 (14.18)	99,164 (13.59)	18,124 (15.53)	<0.0001
DM	1270 (0.67)	13,856 (1.02)	11,239 (1.77)	23,600 (3.24)	8347 (7.15)	<0.0001
DM vintage	0.62 ± 1.8	0.86 ± 2.01	0.91 ± 1.97	0.91 ± 1.9	0.91 ± 1.82	<0.0001
HTN	4911 (2.58)	62,781 (4.62)	54,608 (8.61)	110,487 (15.15)	35,531 (30.44)	<0.0001
Dyslipid	3279 (1.72)	49,198 (3.62)	50,609 (7.98)	95 982 (13.16)	21,898 (18.76)	<0.0001
Weight (kg)	47.82 ± 4.88	59.05 ± 7.41	69.96 ± 6.5	79.18 ± 7.75	94.21 ± 9.71	<0.0001
Height(cm)	164.54 ± 7.51	167.52 ± 8.34	170.69 ± 7.67	171.63 ± 7.26	171.33 ± 7.69	<0.0001
WC (cm)	65.05 ± 5.07	72.97 ± 6.1	80.7 ± 5.14	87.04 ± 5.81	97.38 ± 7.35	<0.0001
BMI	17.62 ± 0.73	20.96 ± 1.25	23.96 ± 0.57	26.83 ± 1.32	32.05 ± 2	<0.0001
SBP(mmHg)	110.26 ± 11.23	115.02 ± 11.83	120.11 ± 11.83	123.98 ± 12.28	129.77 ± 13.7	<0.0001
DBP(mmHg)	69.5 ± 8.2	72.1 ± 8.58	75.27 ± 8.67	77.96 ± 9.06	82.01 ± 10.05	<0.0001
PP(mmHg)	40.76 ± 7.53	42.92 ± 7.88	44.85 ± 8.11	46.01 ± 8.32	47.76 ± 8.97	<0.0001
Glu(mg/dL)	87.29 ± 11.5	89.02 ± 12.78	91.58 ± 15.1	94.21 ± 18.18	98.68 ± 24.84	<0.0001
TC (mg/dL)	171.49 ± 28.02	178.74 ± 30.52	189.91 ± 33.27	198.62 ± 35.09	205.16 ± 36.52	<0.0001
CCI	0.33 ± 0.65	0.3 ± 0.64	0.31 ± 0.65	0.33 ± 0.7	0.41 ± 0.81	<0.0001
CCI grade						<0.0001
0	142,556 (74.89)	1,040,172 (76.47)	486,174 (76.67)	550,174 (75.42)	84,340 (72.27)	
1	37,173 (19.53)	251,251 (18.47)	114,780 (18.1)	135,796 (18.61)	22 901 (19.62)	
2	8 170 (4.29)	51,861 (3.81)	24,345 (3.84)	30,488 (4.18)	5996 (5.14)	
3	2447 (1.29)	16,946 (1.25)	8 792 (1.39)	13,052 (1.79)	3 470 (2.97)	
**** GFR	99.86 ± 49.19	97.33 ± 49.08	95.34 ± 53.58	94.43 ± 54.21	94.85 ± 49.75	<0.0001
CKD event	179 (0.09)	1 854 (0.14)	1235 (0.19)	2107 (0.29)	478 (0.41)	<0.0001

Abbreviations. DM, diabetes mellitus; HTN, hypertension; WC, waist circumference; BMI, body mass index; SBP, systolic blood pressure; DBP diastolic blood pressure; PP, pulse pressure; TC, total cholesterol; GFR, glomerular filtration rate (mL/min/1.73 m^2^); CCI, Charlson Comorbidity Index; CKD, chronic kidney disease. * Alcohol consumptions ≥30 g/day, ** Regular exercise: mid-term exercise ≥5 days or vigorous exercise ≥3 days in a week, *** low income 25%, **** GFR: mL/min/1.73 m^2^.

**Table 3:** Baseline characteristics of participants by level of waist circumference.

Variable	Distribution of Waist Circumference					
	<70/<65 (*n* = 227,355)	70~79/65~74 (*n* = 1,228,295)	80~89/75~84 (*n* = 1,122,351)	90~99/85~94 (*n* = 338,656)	>100/>95 (*n* = 64,227)	*p*
Age	29.57 ± 5.03	31.23 ± 4.94	32.65 ± 4.48	33.02 ± 4.29	32.33 ± 4.39	<0.0001
Age grade						<0.0001
20 s	149,795 (54.01)	484,035 (39.41)	300,697 (26.79)	78,107 (23.06)	18,030 (28.07)	
30 s	127,560 (45.99)	744,260 (60.59)	821,654 (73.21)	260,549 (76.94)	46,197 (71.93)	
Sex (male)	77,960 (28.11)	683,983 (55.69)	924,402 (82.36)	292,422 (86.35)	51,712 (80.51)	
Smoking						<0.0001
None	210,802 (76)	729,199 (59.37)	452,825 (40.35)	118,138 (34.88)	24,218 (37.71)	
Ex-	13,630 (4.91)	120,810 (9.84)	179,006 (15.95)	55,228 (16.31)	8712 (13.56)	
Current	52,923 (19.08)	378,286 (30.8)	490,520 (43.7)	165,290 (48.81)	31,297 (48.73)	
Drinking						<0.0001
None	138,917 (50.09)	498,087 (40.55)	337,093 (30.03)	95,677 (28.25)	21,271 (33.12)	
Mild	129,203 (46.58)	649,434 (52.87)	658,175 (58.64)	194,013 (57.29)	33,538 (52.22)	
Heavy *	9235 (3.33)	80,774 (6.58)	127,083 (11.32)	48,966 (14.46)	9418 (14.66)	
Exercise **	25,947 (9.36)	169,998 (13.84)	176,370 (15.71)	50,108 (14.8)	9200 (14.32)	<0.0001
Income ***	46,573 (16.79)	202,423 (16.48)	149,534 (13.32)	42,585 (12.57)	9096 (14.16)	<0.0001
DM	1597 (0.58)	12,569 (1.02)	24,439 (2.18)	14,423 (4.26)	5284 (8.23)	<0.0001
DM vintage	0.71 ± 1.86	0.83 ± 1.96	0.9 ± 1.95	0.92 ± 1.89	0.97 ± 1.88	<0.0001
HTN	7325 (2.64)	58822 (4.79)	115602 (10.3)	65,827 (19.44)	20,742 (32.29)	<0.0001
Dyslipid	5595 (2.02)	46082 (3.75)	103735 (9.24)	52,556 (15.52)	12,998 (20.24)	<0.0001
Weight (Kg)	50.24 ± 5.99	59.73 ± 7.85	72.03 ± 8.35	83.39 ± 9.21	95.81 ± 11.66	<0.0001
Height (cm)	162.88 ± 7.21	167.15 ± 7.9	171.42 ± 7.36	173.09 ± 7.34	173.33 ± 7.98	<0.0001
WC (cm)	63.13 ± 3.24	72.59 ± 4.07	82.91 ± 3.48	92.47 ± 3.15	103.16 ± 4.56	<0.0001
BMI	18.9 ± 1.54	21.32 ± 1.9	24.48 ± 2.14	27.81 ± 2.37	31.85 ± 3.06	<0.0001
SBP (mmHg)	110.9 ± 11.31	115.41 ± 11.87	121.03 ± 12.15	125.46 ± 12.85	129.82 ± 14.25	<0.0001
DBP (mmHg)	69.82 ± 8.22	72.34 ± 8.59	75.94 ± 8.9	79.02 ± 9.51	82.05 ± 10.49	<0.0001
PP (mmHg)	41.08 ± 7.59	43.08 ± 7.91	45.1 ± 8.18	46.44 ± 8.5	47.77 ± 9.12	<0.0001
Gluco (mg/dL)	87.34 ± 11.1	89.11 ± 12.72	92.29 ± 15.98	95.56 ± 20.34	99.48 ± 26.86	<0.0001
TC (mg/dL)	173.4 ± 28.44	179.07 ± 30.68	191.92 ± 33.91	201.7 ± 35.95	206.67 ± 37.45	<0.0001
CCI	0.31 ± 0.64	0.3 ± 0.63	0.32 ± 0.67	0.36 ± 0.74	0.44 ± 0.85	<0.0001
CCI grade						<0.0001
0	210,942 (76.05)	942,618 (76.74)	854,069 (76.1)	250,141 (73.86)	45,646 (71.07)	
1	51,946 (18.73)	224,591 (18.28)	206,867 (18.43)	65,576 (19.36)	12,921 (20.12)	
2	11,063 (3.99)	46,202 (3.76)	44,407 (3.96)	15,722 (4.64)	3466 (5.4)	
3	3404 (1.23)	14,884 (1.21)	17,008 (1.52)	7217 (2.13)	2194 (3.42)	
GFR ****	98.19 ± 46.4	96.96 ± 48.24	95.26 ± 53.52	95.44 ± 57.64	97.19 ± 55.7	<0.0001
CKD event	305 (0.11)	1785 (0.15)	2439 (0.22)	1089 (0.32)	235 (0.37)	<0.0001

Abbreviations. DM, diabetes mellitus; HTN, hypertension; WC, waist circumference; BMI, body mass index; SBP, systolic blood pressure; DBP diastolic blood pressure; PP, pulse pressure; TC, total cholesterol; GFR, glomerular filtration rate (mL/min/1.73 m^2^); CCI, Charlson Comorbidity Index; CKD, chronic kidney disease. * Alcohol consumptions ≥ 30 g/day, ** Regular exercise: mid-term exercise ≥5 days or vigorous exercise ≥3 days in a week, *** low income 25%, **** GFR:mL/min/1.73 m^2^.

**Table 4:** Multivariate cox analysis for incident chronic kidney disease (CKD) by level of body mass index in young adults.

Group	Total (*n*)	CKD (n)	%	HR (95% Confidence Interval)			
				Model 1	Model 2	Model 3	Model 4
Body mass index 5 level							
<18.5	190,346	179	0.09	0.690 (0.592–0.804)	0.863 (0.740–1.007)	0.876 (0.751–1.023)	0.901 (0.771–1.052)
18.5~23	1,360,230	1854	0.14	1(Ref.)	1(Ref.)	1(Ref.)	1(Ref.)
23~25	634,091	1235	0.19	1.43 (1.330–1.537)	1.278 (1.187–1.376)	1.269 (1.179–1.367)	1.138 (1.057–1.227)
25~30	729,510	2107	0.29	2.122 (1.994–2.259)	1.830 (1.713–1.955)	1.84 (1.722–1.965)	1.368 (1.277–1.465)
30~	116,707	478	0.41	3.013 (2.724–3.332)	2.816 (2.540–3.120)	2.855 (2.576–3.163)	1.550 (1.391–1.727)
*p* for trend				<0.0001	<0.0001	<0.0001	<0.0001
Obesity (Body mass index ≥25)							
<25	2,184,667	3268	0.15	1(Ref.)	1(Ref.)	1(Ref.)	1(Ref.)
≥25	846,217	2585	0.31	2.045 (1.942–2.154)	1.789 (1.695–1.888)	1.802 (1.707–1.902)	1.323 (1.25,0–1.400)
*p* for trend				<0.0001	<0.0001	<0.0001	<0.0001

Abbreviations. CKD, chronic kidney disease; HR, hazard ratio. Model 1: non-adjusted. Model 2: adjusted for age, sex. Model 3: adjusted for model 2 plus smoking, alcohol drinking, regular exercise, income. Model 3: adjusted for model 3 plus glomerular filtration rate, hypertension, diabetes mellitus, dyslipidemia.

**Table 5:** Multivariate cox analysis for incident CKD by level of waist circumference in young adults.

Group	Total (*n*)	CKD (*n*)	%	HR (95% Confidence Interval)			
				Model 1	Model 2	Model 3	Model 4
**Waist circumference 5 level**							
<70/<65	277,355	305	0.11	0.756 (0.67–0.854)	0.95 (0.841–1.074)	0.959 (0.848–1.084)	0.959 (0.848–1.085)
70~79/65~74	1,228,295	1785	0.15	1(Ref.)	1(Ref.)	1(Ref.)	1(Ref.)
70~79/65~74	1,122,351	2439	0.22	1.496 (1.408–1.591)	1.272 (1.194–1.355)	1.281 (1.203–1.365)	1.117 (1.047–1.191)
70~79/65~74	338,656	1089	0.32	2.217 (2.056–2.391)	1.810 (1.674–1.957)	1.858 (1.718–2.009)	1.297 (1.195–1.407)
>100/>95	64,227	235	0.37	2.524 (2.203–2.893)	2.268 (1.977–2.602)	2.334 (2.034–2.677)	1.277 (1.107–1.472)
***p*** **for trend**				<0.0001	<0.0001	<0.0001	<0.0001
**Abdominal obesity (male WC ≥ 90, female WC ≥ 85)**							
No	2,628,001	4529	0.17	1(Ref.)	1(Ref.)	1(Ref.)	1(Ref.)
Yes	402,883	1324	0.33	1.910 (1.796–2.031)	1.642 (1.543–1.748)	1.679 (1.577–1.787)	1.208 (1.132–1.290)
*p* for trend				<0.0001	<0.0001	<0.0001	<0.0001

Abbreviations. CKD, chronic kidney disease; HR, hazard ratio. Model 1: non-adjusted. Model 2: adjusted for age and sex. Model 3: adjusted for model 2 plus smoking, alcohol drinking, regular exercise, income. Model 3: adjusted for model 3 plus glomerular filtration rate, hypertension, diabetes mellitus, dyslipidemia.

**Table 6:** Multivariate cox analysis for chronic kidney disease risk by obesity composite.

Obesity	Abdominal Obesity	Total	Male	Female	Age 20–29 years	Age 30–39 years
No	No (*n* = 2,148,970; 70.9%)	1(Ref.)	1(Ref.)	1(Ref.)	1(Ref.)	1(Ref.)
	Yes (*n* = 35,697; 1.2%)	1.227 (0.979–1.538)	1.204 (0.915–1.584)	1.163 (0.781–1.731)	1.478 (0.698–3.129)	1.203 (0.949–1.524)
Yes	No (*n* = 479,031; 15.8%)	1.318 (1.232–1.409)	1.355 (1.258–1.459)	1.062 (0.895–1.261)	1.217 (0.968–1.531)	1.324 (1.234–1.420)
	Yes (*n* = 367,186; 12.1%)	1.339 (1.247–1.438)	1.356 (1.254–1.467)	1.196 (0.996–1.436)	1.502 (1.190–1.895)	1.320 (1.225–1.423)

Abbreviations. Adjusted for age and sex. Income, diabetes mellitus, dyslipidemia, hypertension, smoking, alcohol drinking, regular exercise, glomerular filtration rate.

## Data Availability

The data presented in this study are available with permission from the National Health Insurance Service review board.

## Supplementary Materials

The following are available online at https://www.mdpi.com/2077-0383/10/5/1065/s1, Table S1: Medication rate for diabetes mellitus, hypertension and dyslipidemia.
