# Supplementary material for: Obesity, Abdominal Obesity and Chronic Kidney Disease in Young Adults: A Nationwide Population-Based Cohort Study
Source: J Clin Med. 2021 Mar 4;10(5):1065. doi: 10.3390/jcm10051065 (PMC7962022; doi:10.3390/jcm10051065)
Supplement: Supplementary file 1 [file jcm-10-01065-s001.pdf]

Supplementary Table 1. Medication rate for diabetes mellitus, hypertension and dyslipidemia

Group	Total (N=3 030 884)	None CKD (N=3 025 031)	CKD (N=5 853)	*P* value
Diabetes mellitus	15631 (0.52)	15360 (0.51)	271 (4.63)	<.0001
Hypertension	51050 (1.68)	50145 (1.66)	905 (15.46)	<.0001
Dyslipidemia	41513 (1.37)	41015 (1.36)	498 (8.51)	<.0001

Variable	Distribution of body mass index					
	<18.5 (N=190 346)	18.5~23 (N=1 360 230)	23~25 (N=634 091)	25~30 (N=729 510)	30~ (N=116 707)	*P*
Diabetes mellitus	187 (0.1)	2922 (0.21)	2811 (0.44)	6815 (0.93)	2896 (2.48)	<.0001
Hypertension	529 (0.28)	8907 (0.65)	9587 (1.51)	23054 (3.16)	8973 (7.69)	<.0001
Dyslipidemia	396 (0.21)	6966 (0.51)	8516 (1.34)	19525 (2.68)	6110 (5.24)	<.0001

Variable	Distribution of waist circumferences					
	<70/<65 (N=227 355)	70~79/65~74 (N=1 228 295)	80~89/75~84 (N=1 122 351)	90~99/85~94 (N=338 656)	>100/>95 (N=64 227)	*P*
Diabetes mellitus	268 (0.1)	2667 (0.22)	6351 (0.57)	4400 (1.3)	1945 (3.03)	<.0001
Hypertension	878 (0.32)	8452 (0.69)	21649 (1.93)	14575 (4.3)	5496 (8.56)	<.0001
Dyslipidemia	641 (0.23)	6602 (0.54)	19002 (1.69)	11621 (3.43)	3647 (5.68)	<.0001
